# Editorial: Metabolic engineering for bioresources and bioenergies production from microalgae

**DOI:** 10.3389/fbioe.2022.1114854

**Published:** 2023-01-05

**Authors:** Fantao Kong, Xupeng Cao, Miriam Schulz-Raffelt

**Affiliations:** ^1^ School of Bioengineering Dalian University of Technology, Dalian, China; ^2^ Dalian Institute of Chemical Physics Chinese Academy of Sciences, Dalian, China; ^3^ Molekulare Biotechnologie and Systembiologie, TUKaiserslautern, Kaiserslautern, Germany

**Keywords:** metabolic engineering, metabolites, bioresource, bioenergy, microalgae

Microalgae are a group of photosynthetic microorganisms that can utilize atmospheric CO_2_ as a carbon source. They are regarded as promising green cell factories for the production of biofuels and highly valuable products. However, the production of biofuels and other valuable products is currently not economically viable in microalgae, due to the low yield. Therefore, rational metabolic engineering of microalgae to obtain robust microalgal strains is required, and a comprehensive understanding of lipid metabolism is also essential. Moreover, state-of-the-art molecular genetic tools including synthetic biological strategies will also facilitate potentiation of microalgae as cell factories for bioresource and bioenergy production. Thus, it seems timely to launch this Research Topic to collect recent achievements on metabolic or process engineering in microalgae that can enhance the production of biofuels and other valuable products.

**FIGURE 1:**
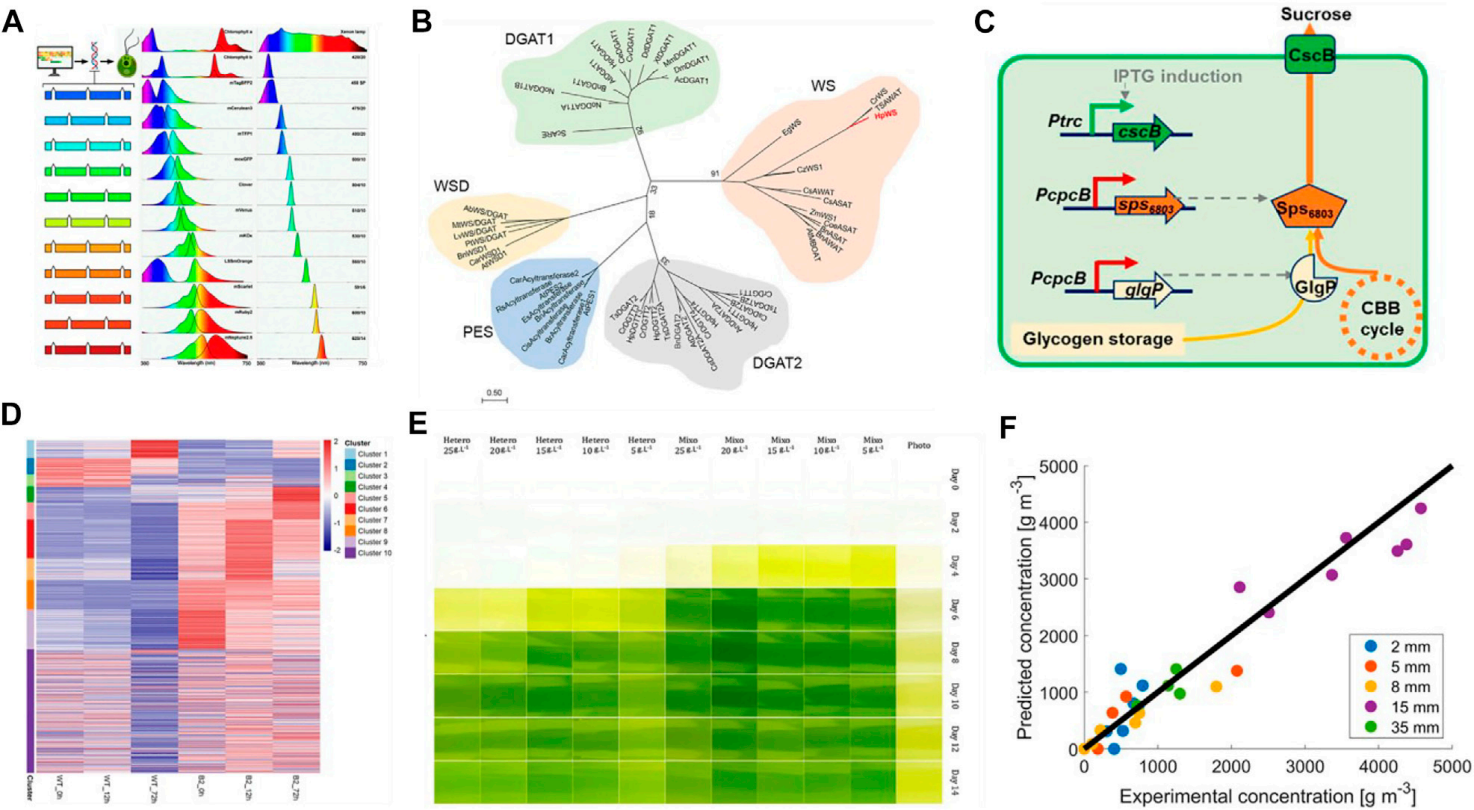
Representative articles in this Research Topic. **(A)** Technical advances for transformant selection in *C. reinhardtii* (Gutiérrez et al.). **(B)** Novel bifunctional wax ester synthase (HpWS), identified in *H. pluvialis*, and heterogeneous expression of *HpWS* in *C. reinhardtii* for triacylglycerol accumulation (Ma et al.). **(C)** Overexpression of *GlgP* (glycogen phosphorylase) increased sucrose production in *S. elongatus* (Dan et al.). **(D)** Proteomic analysis of dark-adapted *E. gracilis* and the bleached mutant against light stimuli (Chen et al.) **(E)** Effects of different cultivation modes on the biomass productivity and biocomponent contents in *Chlorella* sp (Yun et al.). **(F)** Self-shading effects on microalgal growth during continuous cultivation in photobioreactors (Saccardo et al.).

This Research Topic includes eight research articles/original reviews on microalgal biology and biotechnology. These studies include strategic transgene selection, enzyme engineering, proteomic analysis, cultivation mode comparation, and mathematical modeling of growth. In addition, one review discussed the synthetic biology perspective on bioengineering tools for microalgae. Fluorescent proteins (FP) are useful reporters to monitor gene expression and subcellular localization. Gutiérrez et al. Demonstrated that a broad set of optimized synthetic FP can be used as fusion partners to monitor transgene expression in the eukaryotic model microalga *Chlamydomonas reinhardtii* (Figure 1A). This technical advance will facilitate high-throughput screening of algal transformants to identify high transgenes expression or trait stacking cells, since the target recombinant-FP fusion protein can be visualized *in situ* after electrophoresis. In the green microalga *Haematococcus pluvialis*, a novel bifunctional wax ester synthase (HpWS), involved in early triacylglycerol (TAG) accumulation under high light conditions (HL), was identified (Figure 1B) by Ma et al. Heterologous expression of *HpWS* in TAG-deficient *Saccharomyces cerevisiae* can restore the capability of wax and TAG biosynthesis. In addition, the overexpression of *HpWS* in *Chlamydomonas reinhardtii* enhanced TAG production (Ma et al.) This study provides a new molecular target for genetic manipulation to increase TAG and wax accumulation in microalgae. Dan et al. reported that overexpression of *GlgP* (glycogen phosphorylase) promoted effective glycogen degradation and increased sucrose production in the prokaryotic microalga *Synechococcus elongatus* (Figure 1C). This study avoided physiological and metabolic impairments caused by targeting of the glycogen synthesis pathway and provided novel insights into metabolic engineering approaches for efficient photosynthetic biosynthesis. Chen et al. investigated the proteomic responses of the dark-adapted unicellular eukaryotic microalga *Euglena gracilis* (*Euglena*) and the bleached mutant against light stimuli (Figure 1D). This study provided useful information about the unknown functions of residual plastids in bleached *Euglena* mutants. In addition, the same group reviewed the progress of molecular genetic engineering approaches in *Euglena*. The authors summarized various methods for transgene delivery, gene silencing, and genome editing, such as overexpression, RNA interference, and CRISPR/Cas9 systems (Chen et al.). The tools and knowledge summarized will pave the way for developing a synthetic biological approach to produce useful bioproducts in *Euglena*. Microalgae can utilize both organic and inorganic carbon sources to power their growth. The effects of different cultivation modes on the biomass productivity and biocomponent contents in *Chlorella* sp. Were examined by Yun et al. The authors found that mixotrophic cultivation of *Chlorella* sp. Can improve the yields of biomass, lipid, protein, and pigment in comparison to photoautotrophic and heterotrophic conditions (Figure 1E) (Yun et al.). Interestingly, this team also found that microbial communities composed of algae improved biofloc technology, including the nitrogen-related material cycle in *Litopenaeus vannamei* farms (Yun et al.). To realize large-scale cultivation of microalgae, photobioreactors have been commonly used. The self-shading effects on microalgal growth during continuous cultivation in photobioreactors were studied by Saccardo et al. The authors found that a strong shading effect occurs when the thicknesses of the algae is above 8 mm. To highlight the effect of mixing cycles, the study proposed an analogy between small-scale operations using continuous light and photobioreactors using pulsed light, based on characteristic parameters obtained from mathematical modeling of the growth (Figure 1F) (Saccardo et al.).

The articles in this Research Topic demonstrate that strategies such as synthetic biology, metabolic engineering, proteomic analysis, and process engineering will contribute to the production of biofuels and other valuable biocompounds from microalgae at industrial levels.

